# Evolution‐Guided Unidirectional Counter‐Clockwise Rotation for Nonretractable Screw‐in Lead Removal

**DOI:** 10.1002/joa3.70300

**Published:** 2026-02-16

**Authors:** Masatsugu Nozoe, Takafumi Sakamoto, Satoshi Tsujioka, Daisuke Nagatomo, Nobuhiro Suematsu, Toru Kubota

**Affiliations:** ^1^ Division of Cardiology, Cardiovascular and Aortic Center, Saiseikai Fukuoka General Hospital Fukuoka Japan

**Keywords:** evolution mechanical sheath, lead extraction, myocardial perforation, screw‐in lead, unidirectional rotation

## Abstract

Unidirectional counter‐clockwise manipulation of the Evolution RL sheath after adequate dissection enables controlled lead body rotation and gradual unscrewing. This technique may reduce myocardial injury during non‐retractable screw‐in lead removal.

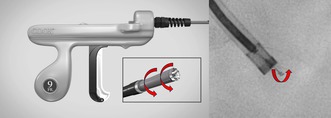

Retractable screw‐in leads are generally considered less risky to extract than passive tined leads [1]. Failure to retract the screw markedly increases the risk of myocardial injury, particularly when the lead is positioned at a non‐septal site such as the right atrial appendage [2]. In cases of suspected perforation involving a nonretractable screw lead, conventional counter‐traction may cause myocardial tearing, increasing the risk of complications during percutaneous extraction [3]. We herein describe a potentially safer extraction technique—evolution‐guided unidirectional counterclockwise rotation—which enables controlled screw‐out by body turn without excessive traction under appropriate conditions.

A 71‐year‐old man with Brugada syndrome had received a dual‐coil implantable cardioverter‐defibrillator (ICD) lead (ISOLINE2CR‐6, MicroPort CRM, Clamart, France) 13 years earlier. He was referred for transvenous lead extraction because of gradually rising impedance and an increased pacing threshold. Computed tomography and right ventriculography raised suspicion of right ventricular (RV) lead‐tip perforation beyond the myocardial border (Figure 1). The ISOLINE 2CR‐6 lead employs a manufacturer‐specific screw‐in fixation mechanism that requires a dedicated stylet, which is currently unavailable. The presence of myocardial perforation combined with a nonretractable RV screw made percutaneous extraction particularly high risk.

**FIGURE 1:**
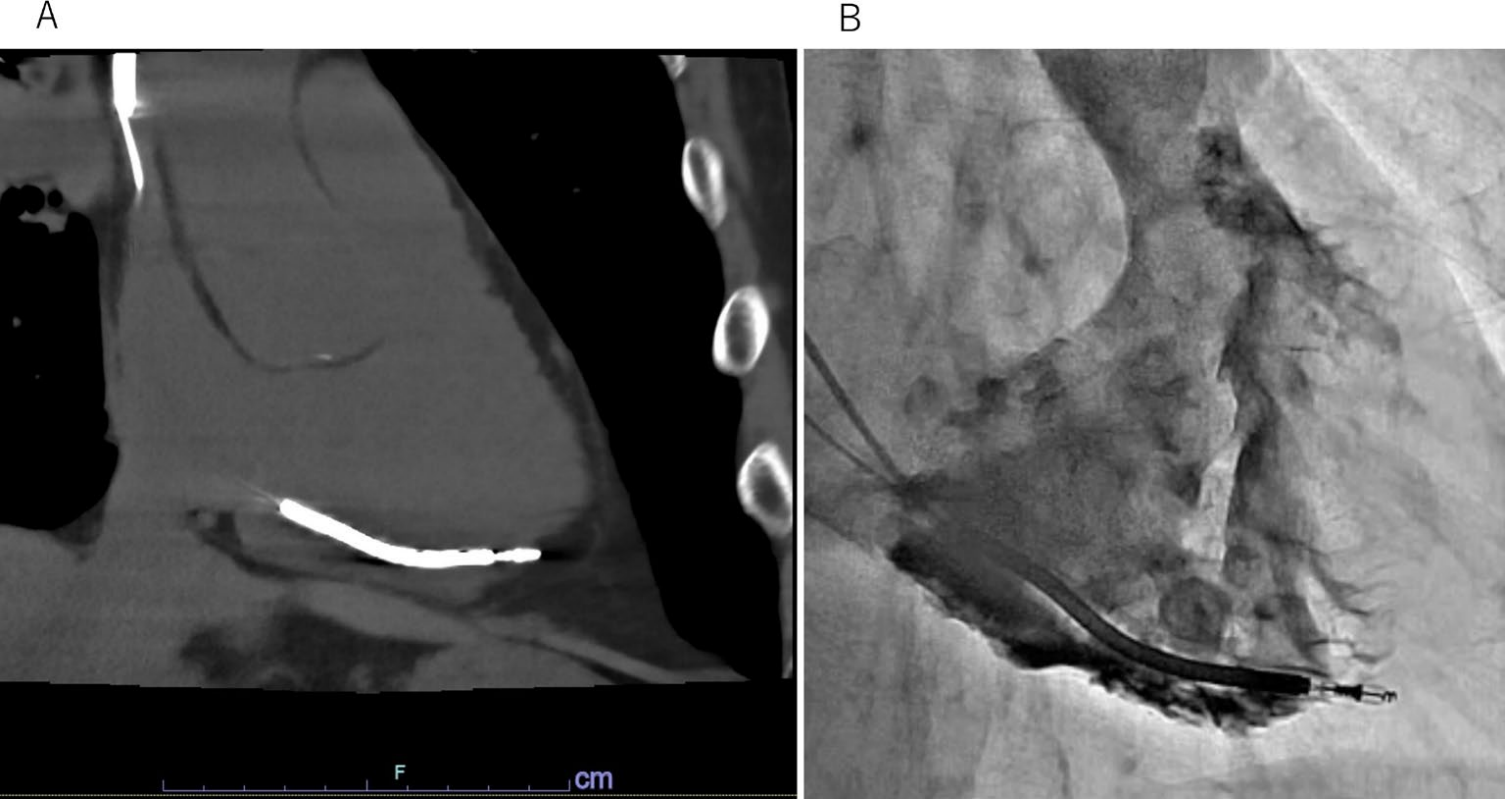
Computed tomography (A) and right ventriculography (B) suggest right ventricular (RV) lead‐tip perforation beyond the myocardial border.

The procedure was performed under general anesthesia in a hybrid operating room with surgical standby and continuous transesophageal echocardiographic (TEE) monitoring. The RV lead was grasped with a Needle's Eye Snare (Merit Medical Systems, South Jordan, UT, USA) via the femoral vein to minimize mechanical stress on the suspected perforation site. Most adhesions were dissected using an 11‐Fr Evolution RL mechanical sheath (Merit Medical) in combination with a SteadySheath (Merit Medical). Because the adhesion was especially dense around the RV shock coil, the sheath was advanced to a 16‐Fr GlideLight laser sheath (Philips, Amsterdam, The Netherlands). Consequently, the dissection was extended to the myocardial surface. Before applying counter traction, the 16‐Fr GlideLight laser sheath was replaced with an 11‐Fr Evolution RL mechanical sheath to release the active‐fixation helix safely. The SteadySheath was advanced to the dissected myocardial surface; however, the 11‐Fr Evolution RL mechanical sheath could not be advanced beyond the site previously dissected using the 16‐Fr GlideLight laser sheath. Unidirectional counterclockwise body rotation was performed at that location. The 11‐Fr Evolution RL mechanical sheath was manipulated to rotate only in the counterclockwise direction without counter‐traction (Figure 2). Evolution‐guided unidirectional counterclockwise body rotation resulted in gradual, unidirectional rotation of the lead body, allowing progressive unscrewing of the helix from the myocardium (Figure 3). Complete release was achieved without myocardial injury (Figure 4), and TEE confirmed the absence of pericardial effusion throughout the procedure.

**FIGURE 2:**
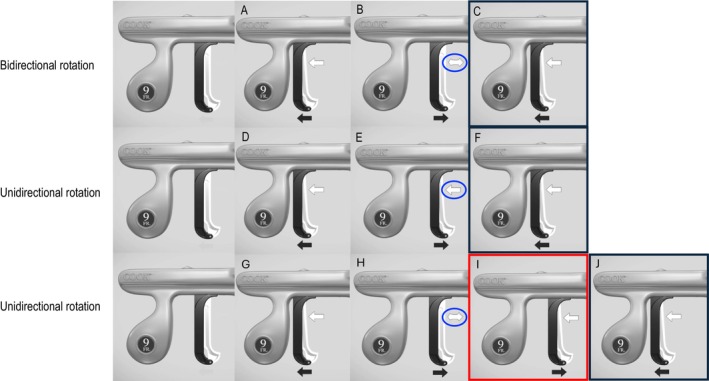
How to manipulate the Evolution RL mechanical sheath for unidirectional counterclockwise rotation. Rotate the inner Evolution RL sheath by squeezing and releasing the trigger activation handle. For bidirectional rotation, squeeze the trigger to activate sheath rotation (A). Returning the trigger to its forward‐most position (B) mechanically changes the rotational direction of the sheath to the opposite of the previous rotation (C). For unidirectional rotation, squeeze the trigger to activate sheath rotation (D) and repeat without allowing the trigger to return to its forward‐most position (E, F). In this mode, only the black trigger is released while the white trigger remains engaged, maintaining the same rotation direction. Alternatively, if the trigger is returned to its forward‐most position (H), the rotation direction is mechanically reversed. In this situation, a slight tap of the white trigger only, without squeezing the black trigger, changes the rotational direction again to the opposite side (I), allowing the operator to maintain unidirectional control (J).

**FIGURE 3:**
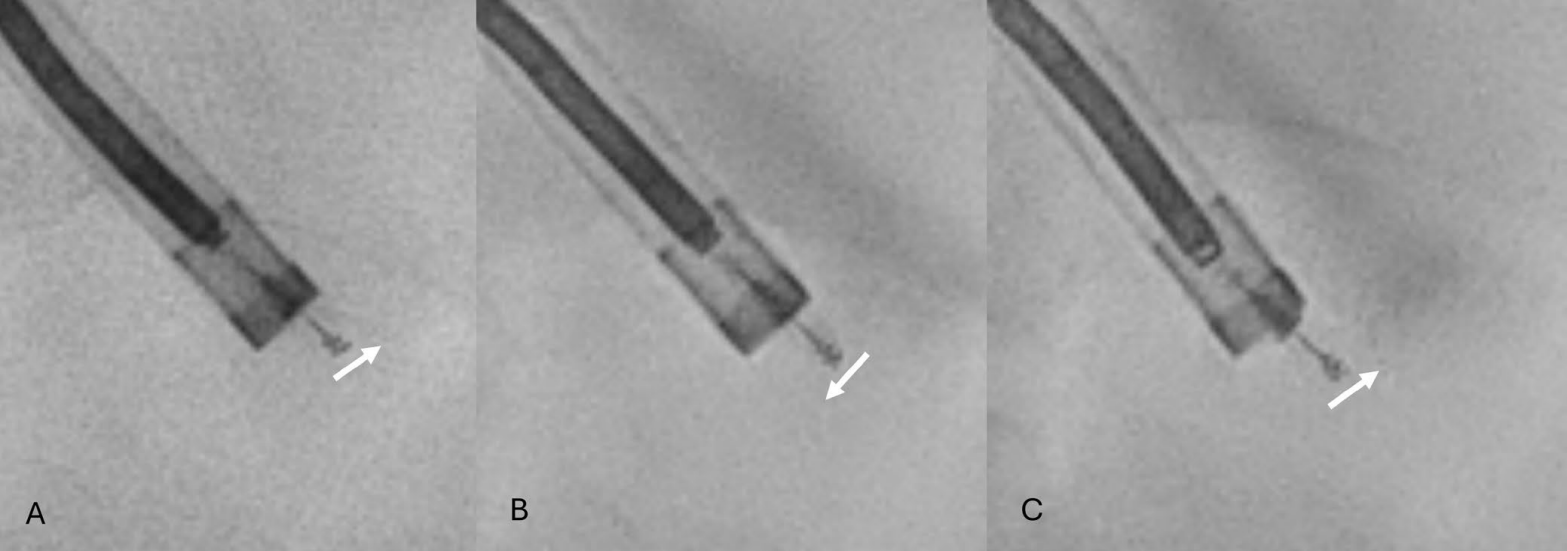
Sequential enlarged fluoroscopic images demonstrate gradual unidirectional rotation of the lead body during Evolution‐guided unidirectional counterclockwise rotation. Three static frames (A–C) show progressive changes in the orientation of the screw helix. Arrows indicate the changing orientation of the screw helix, confirming gradual unscrewing from the myocardium.

**FIGURE 4:**
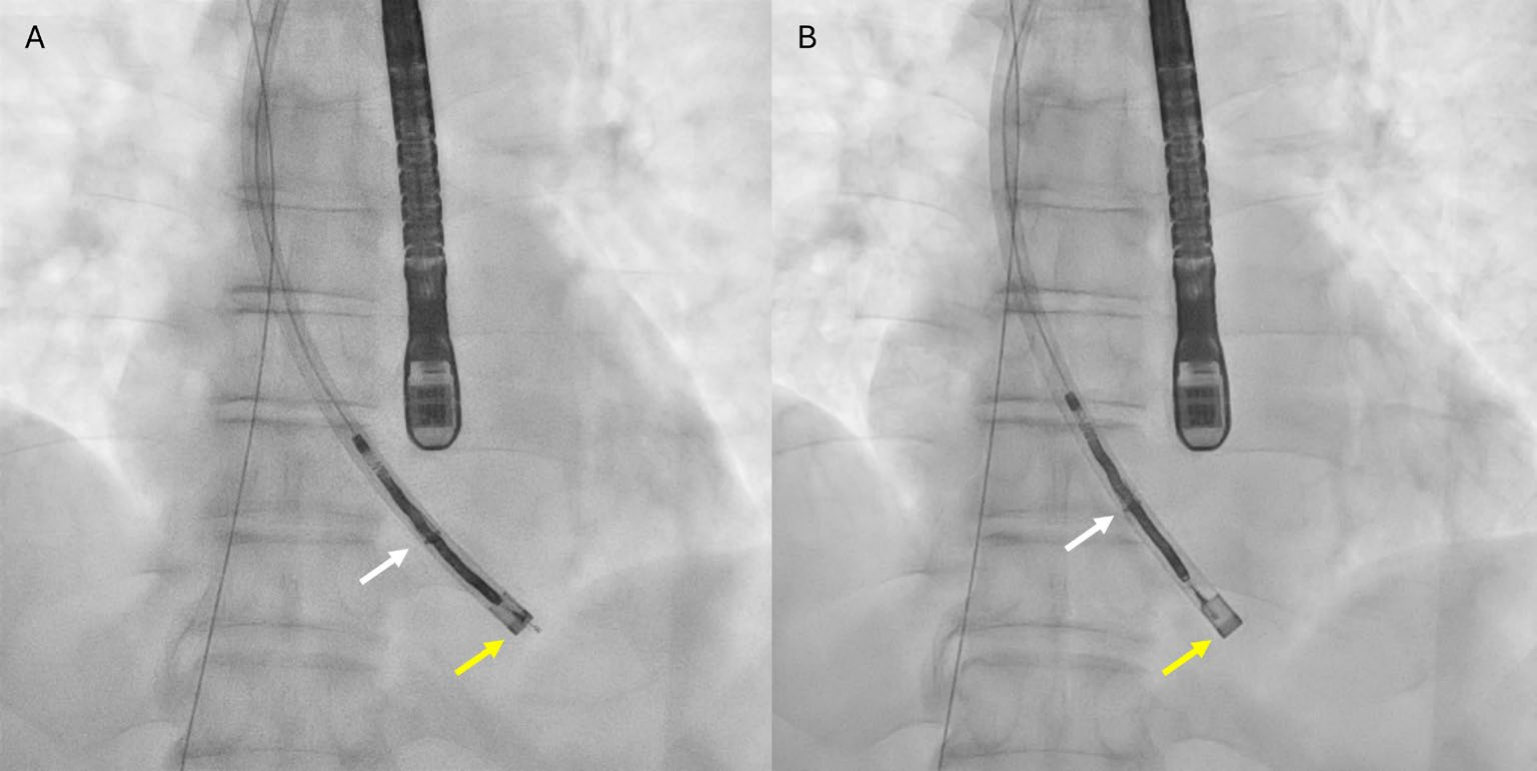
(A) Fluoroscopic image obtained during Evolution‐guided unidirectional counterclockwise rotation for extraction of the right ventricular (RV) nonretractable screw‐in lead with suspected perforation. The SteadySheath was advanced to the dissected myocardial surface; however, the 11‐Fr Evolution RL mechanical sheath could not be advanced beyond the site previously dissected using the 16‐Fr GlideLight laser sheath. Unidirectional counterclockwise body rotation was performed at that location. The distal tip of the SteadySheath and the Evolution RL sheath are indicated by the yellow and white arrows, respectively. The 11‐Fr Evolution RL mechanical sheath was manipulated to rotate only in the counterclockwise direction without counter‐traction. (B) Complete release of the RV screw‐in lead was achieved without myocardial injury.

The same technique was successfully applied in another patient with a cardiac resynchronization therapy defibrillator (CRT‐D), in whom atrial lead damage was identified during generator replacement. The patient underwent urgent lead extraction and reimplantation; however, the screw could not be retracted even when the locking stylet was rotated using Pean forceps. Lead dissection was performed using a 9‐Fr Evolution RL mechanical sheath; however, the procedure was interrupted by dense adhesion between the leads in the superior vena cava. At that site, Evolution‐guided unidirectional counterclockwise rotation was applied to this nonretractable atrial lead (Figure 5A). This technique safely released the screw from the atrial wall without cardiac injury (Figure 5B).

**FIGURE 5:**
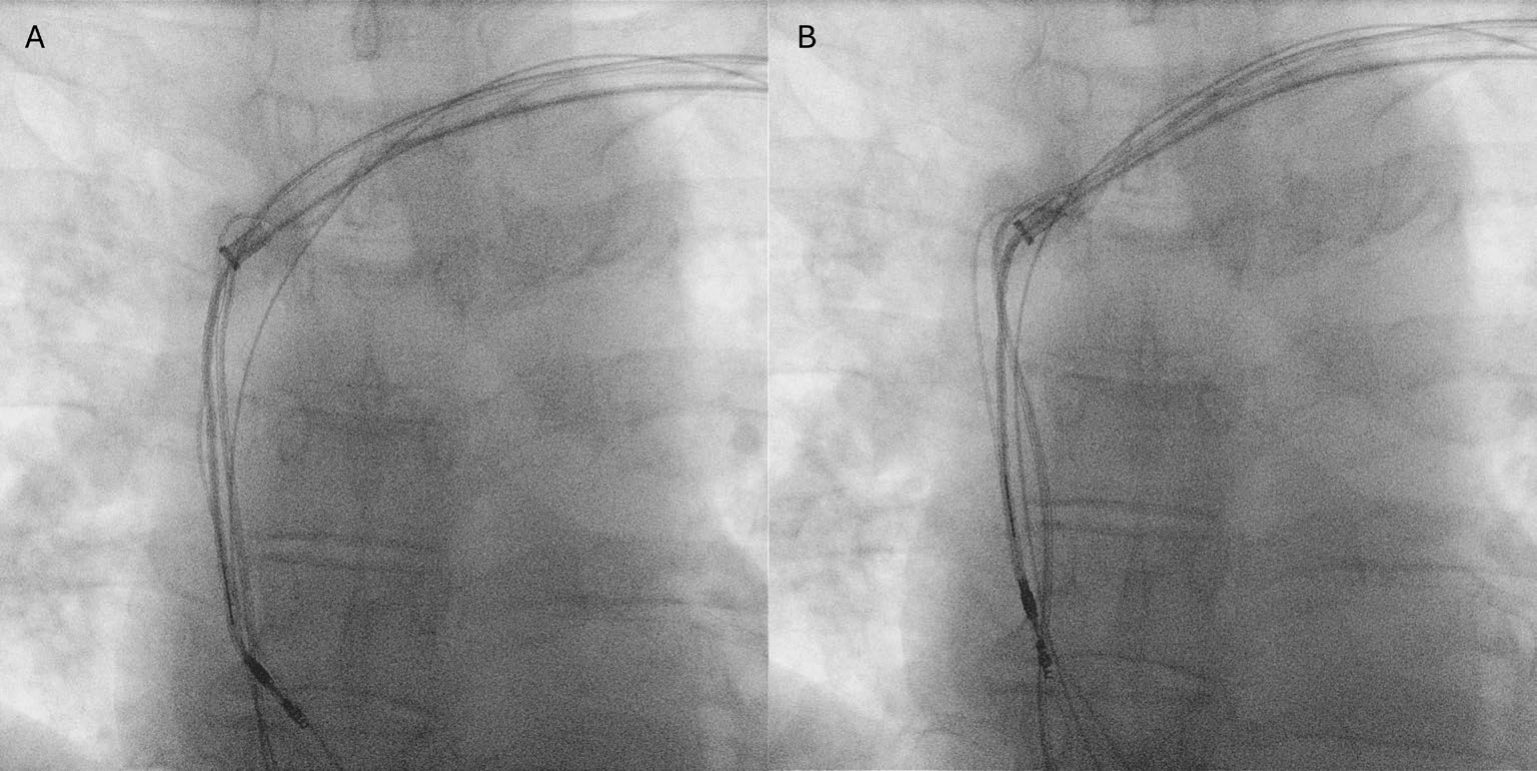
Fluoroscopic images of nonretractable right atrial lead extraction using Evolution‐guided unidirectional counterclockwise body rotation. (A) Evolution‐guided unidirectional counterclockwise rotation was performed at the superior vena cava, where dense adhesion between the leads was present. (B) The atrial screw‐in lead tip was successfully released from the atrial wall without causing myocardial injury.

In nonretractable screw‐in leads, lead body turn has traditionally been used to release a fixed helix while minimizing stress on the myocardial wall. This Evolution‐guided unidirectional counterclockwise rotation technique is based on the same principle using the Evolution sheath to transmit controlled unidirectional torque. Manual counterclockwise rotation of the powered sheath itself could theoretically produce a similar result; however, this maneuver is rarely feasible in clinical settings because a large powered sheath cannot be properly rotate within the vessel. Therefore, attempting this technique before counter‐traction may reduce myocardial injury in some cases with nonretractable screw‐in leads. The present technique is applicable only when appropriate matching of sheath size to the lead, including the degree of surrounding adhesions, allows the lead to rotate in concert with the Evolution RL sheath. Extraction of a 1.5‐year‐old Ingevity pacemaker lead was attempted without lead cutting, and all adhesions except the screw at the myocardium were completely dissected. Although Evolution‐guided unidirectional rotation using an 11‐Fr Evolution RL sheath was attempted under conditions considered favorable for this method, this technique did not work because of a diameter mismatch between the sheath and the lead with minimal surrounding adhesions. In addition, this technique may be effective only after sufficient lead dissection has been achieved to permit reliable torque transmission to the distal screw tip. In some cases, however, the fixation of the lead tip is spontaneously released during adhesion dissection at the proximal site, and in such situations, this technique is not required. Because this technique can be applied only under such specific conditions, it is one of the major limitations of this method. When dense adhesions around the screw helix or lead tip are present, the safety of this technique cannot be fully predicted. Although its feasibility and reliability have not yet been established, this method may help reduce risk under specific conditions and could provide a useful option for challenging cases of lead extraction.

In the second case, complete release of adhesions was not achieved; however, dissection had progressed sufficiently to the superior vena cava to indicate that the lead might rotate, and the technique was therefore attempted at that point. If this technique had failed, additional adhesion dissection would have been required; however, this technique enabled successful extraction without requiring any further dissection of dense adhesions.

In conclusion, evolution‐guided unidirectional rotation provides a simple, controlled, and reproducible method for the safe removal of nonretractable screw‐in leads, particularly in high‐risk situations such as myocardial perforation. Further clinical experience and accumulation of additional cases will be necessary to validate this technique and better define its role.

## Ethics Statement

The procedure was conducted in accordance with institutional ethical standards.

## Consent

Written informed consent was obtained from the patient for publication.

## Conflicts of Interest

The authors declare no conflicts of interest.

## Data Availability

Data sharing not applicable to this article as no datasets were generated or analysed during the current study.
